# RNF213 Polymorphisms in Intracranial Artery Dissection

**DOI:** 10.3390/genes15060725

**Published:** 2024-06-01

**Authors:** Marialuisa Zedde, Ilaria Grisendi, Federica Assenza, Manuela Napoli, Claudio Moratti, Claudio Pavone, Lara Bonacini, Giovanna Di Cecco, Serena D’Aniello, Maria Simona Stoenoiu, Alexandre Persu, Franco Valzania, Rosario Pascarella

**Affiliations:** 1 Neurology Unit, Stroke Unit, Azienda Unità Sanitaria Locale-IRCCS di Reggio Emilia, Viale Risorgimento 80, 42123 Reggio Emilia, Italy; grisendi.ilaria@ausl.re.it (I.G.); assenza.federica@ausl.re.it (F.A.); valzania.franco@ausl.re.it (F.V.); 2 Neuroradiology Unit, Azienda Unità Sanitaria Locale-IRCCS di Reggio Emilia, Viale Risorgimento 80, 42123 Reggio Emilia, Italy; napoli.manuela@ausl.re.it (M.N.); moratti.claudio@ausl.re.it (C.M.); pavone.claudio@ausl.re.it (C.P.); bonacini.lara@ausl.re.it (L.B.); dicecco.giovanna@ausl.re.it (G.D.C.); daniello.serena@ausl.re.it (S.D.); pascarella.rosario@ausl.re.it (R.P.); 3 Department of Internal Medicine, Rheumatology, Cliniques Universitaires Saint-Luc, Université Catholique de Louvain, 1200 Brussels, Belgium; maria.stoenoiu@saintluc.uclouvain.be; 4 Division of Cardiology, Cliniques Universitaires Saint-Luc, Université Catholique de Louvain, 1200 Brussels, Belgium; alexandre.persu@saintluc.uclouvain.be; 5 Pole of Cardiovascular Research, Institut de Recherche Expérimentale et Clinique, Université Catholique de Louvain, 1200 Brussels, Belgium

**Keywords:** RNF213, moyamoya disease, intracranial artery dissection, magnetic resonance angiography, intracranial stenosis, atherosclerosis

## Abstract

The ring finger protein 213 gene (RNF213) is involved in several vascular diseases, both intracranial and systemic ones. Some variants are common in the Asian population and are reported as a risk factor for moyamoya disease, intracranial stenosis and intracranial aneurysms. Among intracranial vascular diseases, both moyamoya disease and intracranial artery dissection are more prevalent in the Asian population. We performed a systematic review of the literature, aiming to assess the rate of RNF213 variants in patients with spontaneous intracranial dissections. Four papers were identified, providing data on 53 patients with intracranial artery dissection. The rate of RNF213 variants is 10/53 (18.9%) and it increases to 10/29 (34.5%), excluding patients with vertebral artery dissection. All patients had the RNF213 p.Arg4810Lys variant. RNF213 variants seems to be involved in intracranial dissections in Asian cohorts. The small number of patients, the inclusion of only patients of Asian descent and the small but non-negligible coexistence with moyamoya disease familiarity might be limiting factors, requiring further studies to confirm these preliminary findings and the embryological interpretation.

## 1. Introduction

The ring finger protein 213 gene (RNF213; NM_001256071.2) encodes a 590 kDa protein containing a RING finger domain with E3 ubiquitin–protein ligase activity and two regions of ATPase-associated domains. RNF213 is related to angiogenesis and vascular inflammation in experiments in vitro and in vivo, but its exact physiologic functions remain unknown [1]. Its role as a vasculopathy–susceptibility locus was demonstrated in 2011 in a cohort of Japanese families with moyamoya disease, investigated through genome-wide linkage analysis, identifying heterozygosity for RNF213 p.Arg4810Lys (c.14429G>A, rs112735431) polymorphism as significantly associated with the disease [2]. Moyamoya disease is an intracranial, non-atherosclerotic, non-inflammatory steno-occlusive progressive disease, characterized by the development of compensatory collateral networks, which give the disease its name [3]. After this first demonstration, a genome-wide association study analysis in Japanese patients with moyamoya disease found that 95% and 73% of familial and non-familial patients, respectively, had the RNF213 p.Arg4810Lys variant, providing an odds ratio of 190.8 for having moyamoya disease [4]. The same association was documented in Korean and Chinese cohorts of moyamoya patients [5,6] with a lower allele frequency for the RNF213 p.Arg4810Lys variant in Chinese patients compared to Japanese and Korean patients, and another variant also being present in Chinese patients (RNF213 p.Ala5021Val) as a susceptibility factor for the development of moyamoya disease [7]. In large cohorts, the RNF213 (c.14576G>A) mutation was reported in 69.9–85.4% of cases of moyamoya disease [8,9,10]. In non-Asian patients, and particularly in Caucasian patients, the RNF213 p.Arg4810Lys variant is extremely rare, and several different pathogenetic variants of RNF213 have been identified [7] with a lower odds ratio (2.24) [11], showing a strong association with moyamoya disease only in familial cases [7].

Additionally, the RNF213 c.14576G>A variant has been implicated in other vascular diseases. The first description was of the pulmonary (peripheral pulmonary artery stenosis with pulmonary hypertension) and coronary arteries [12], which are sometimes associated with moyamoya disease [11,13], particularly in carriers of the homozygous mutation of RNF213, p.Arg4810Lys. In the following years, the concept of RNF213-associated vasculopathy was developed [14]. It has been suggested that heterozygous R4810K variant causes classical moyamoya disease, but the same variant is associated with moyamoya disease and systemic vascular disease when present in the homozygous state, in a gene dosage-dependent manner [11]. Homozygous patients showed a diffuse narrowing of the aorta and iliofemoral arteries, together with stenosis of renal, celiac or peripheral pulmonary arteries, with or without moyamoya disease [15]. However, heterozygous patients were mostly asymptomatic or had isolated moyamoya disease. This association suggests a high penetrance of systemic vasculopathy in homozygous patients and a low penetrance of moyamoya disease in heterozygous patients.

**Figure 1:**
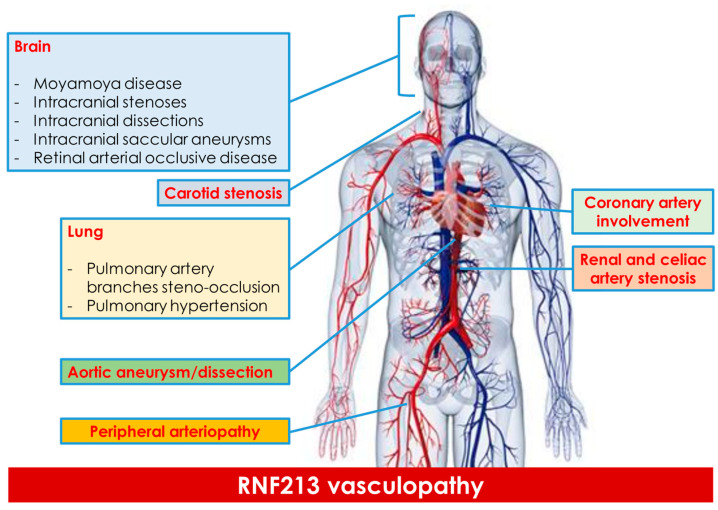
The main sites of systemic vasculopathy described in RNF213 variants are summarized.

Returning to the intracranial arteries, in East Asian patients, the RNF213 (c.14576G>A) variant is implicated in 21–23.2% of intracranial internal carotid artery stenosis (ICS) cases, but not in vertebral artery stenosis [9,16]. This variant is notably prevalent among East Asians without intracranial disease, with allele frequencies of 2.8% in Japanese patients, 2.5% in Korean patients and 1.1% in Chinese patients, while it is rarely found in Western Caucasians [9,17]. The reported Minor Allele Frequency^9^ (MAF) for RNF213 is 0.0012 and it significantly increases the risk of having moyamoya disease in Japanese, Korean and Chinese patients Interestingly, the frequency of RNF213 (c.14576G>A) in Japanese patients with cerebral aneurysms (CAN) is lower than in those with ICS and is similar to that in control subjects without vascular lesions, ranging from 0 to 2.1% [18]. Moreover, RNF213 variants (p.Arg2438Cys and p.Ala2826Thr) have been identified in French–Canadian patients with intracranial aneurysms. However, few studies have explored the relationship between intracranial aneurysms and RNF213, failing to clarify the site, morphology (saccular, fusiform or dissected) and clinical characteristics of these aneurysms [19]. The etiology of intracranial aneurysms varies based on the site of origin within the intracranial vessels, exhibiting different frequencies in men and women, as well as varying rupture rates. Therefore, assessing these aneurysms requires a nuanced approach considering their diverse origins and characteristics [20,21]. Figure 1 summarizes the main sites of arterial diseases associated with RNF213 variants. 

Intracranial artery dissection is a rare neurovascular condition whose genetic underpinnings are less understood compared to extracranial artery dissection [22,23]. Most research involves patients from Asia, with 95% of studies involving more than 40 patients and 61% of studies including 20 to 39 patients focusing on this population [24,25,26,27,28]. Among adults, a higher incidence of intracranial artery dissection is observed in Asian men, a trend not seen in non-Asian populations. The average age at diagnosis is 50.4 years (ranging from 47 to 61 years) [22], with older patients being more likely to present with subarachnoid hemorrhage. The differences in the prevalence and characteristics of intracranial artery dissections between ethnic groups, as well as the higher frequency in children compared to adults, indicate potential genetic risk factors. However, the genetic basis for intracranial artery dissections remains largely unexplored. In this systematic review, we aimed to assess the prevalence of RNF213 variants in intracranial artery dissection in Asian population, as both issues are more prevalent in Asian than in non-Asian people.

## 2. Materials and Methods

A systematic review of the published papers reporting RNF213 variations in patients with acute spontaneous intracranial artery dissection was performed, following the Meta-Analyses and Systematic Reviews of Observational Studies (MOOSE) group guidelines [29]. We searched PubMed and EMBASE databases for studies addressing RNF213 status in spontaneous intracranial artery dissection without lower time limits until 28 February 2024. We used the following keywords for PubMed: “((RNF213) OR (ring finger protein 213))” AND “(intracranial artery dissection)”. We excluded patients with moyamoya disease and traumatic or iatrogenic intracranial artery dissection. In addition, we applied forward and backward citation tracking to improve the results. All studies presenting original data about the topic of the review were included. We limited the selection to English studies and excluded case reports and studies on nonhuman subjects. Abstracts presented at relevant scientific meetings were excluded because of the lack of relevant information. We avoided including duplicated datasets. Two investigators (MZ, RP) independently screened the papers retrieved in the literature search according to the previously detailed criteria. The NIH Quality Assessment Tool for Observational Cohort and Cross-Sectional Studies [30] was used to evaluate each eligible publication. The following information was extracted: authors, year of publication, country, number of patients and main demographic and diagnostic features, RNF213 variants and the rate of their identification. In the case of missing values, we tried to derive them whenever possible [30]. Disagreements between the two reviewers were addressed and resolved by consensus.

## 3. Results

**Figure 2:**
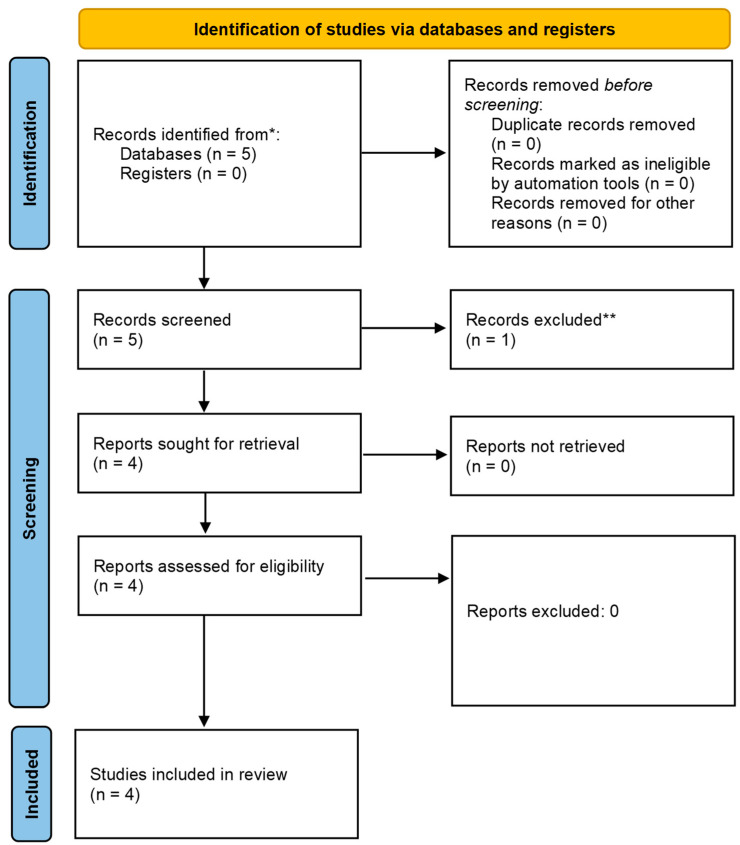
PRISMA diagram [31]. ** The excluded paper [32] included both symptomatic and asymptomatic patients with intracranial artery diseases and relies on a single MRI technique to diagnose the subtype of the disease, and this is not reliable for intracranial artery dissections. Moreover, details on RNF213 polymorphisms were not provided.

The systematic review of the available literature was performed in accordance with the Section 2. The selection of the studies is summarized in the PRISMA diagram [31] (Figure 2).

A total number of four studies were retrieved and analyzed. The citation tracking did not find any other published report about the topic of the review. The results of the data extraction are summarized in Table 1.

The rate of RNF213 variants in intracranial artery dissection is reported as 10/53 (18.9%) and increases to 10/29 (34.5%) when excluding patients with VA dissection. All patients had the RNF213 p.Arg4810Lys variant. 

## 4. Discussion

The RNF213 gene plays a significant role in the ethnic disparity observed in the localization of cerebral vascular diseases. Initially recognized as a susceptibility gene for moyamoya disease [39], RNF213 variants are now linked to a broader spectrum of vascular conditions beyond moyamoya [40], including intracranial atherosclerosis. This association underscores the gene’s involvement in various non-moyamoya vascular diseases, further highlighting its significance in understanding the genetic underpinnings of cerebral vascular disorders [11,16,33,37]. In addition, the RNF213 R4810K variant is associated with smaller intracranial arteries, suggesting impaired vasculogenesis [41,42]. Hongo et al. showed that the outer diameter of the MCA was smaller in R4810K carriers [41]. Moreover, negative remodeling, investigated with high-resolution MRI, involves all the intracranial arteries; the stenotic MCA segments are measured in patients with this variant [42], predisposing the smaller intracranial arteries to hemodynamic compromise. The exact role and mechanisms of RNF213-related vascular impairment are not well known and whether RNF213 mutations induce a loss-of-function or gain-of-function allele status is controversial. Missense mutations might disrupt gene transcription and protein function to a certain extent, causing the pathological dysregulation of substrate ubiquitination due to changes in the functional domain [43,44]. Among the studies suggesting the role of RNF213 in vascular wall construction [2], a functional study on the RNF213 p.Arg4810Lys variant proposed that mutations in the founder are a risk factor for moyamoya disease through reduced angiogenic activity [45,46] and induced mitotic abnormalities [47]. A study on animal models found a link to a variety of artery-wall developments, showing thinning of the intima and media layers after ligation of the common carotid artery in RNF213-knockout mice [48], thinning in vascular walls and increased Mmp9 expression [49] or enhanced post-ischemic angiogenesis [50]. Moreover, RNF213 was associated with inflammatory responses and angiogenesis [45,51]. All these studies show an association between RNF213 and vascular remodeling processes, even the formation of an aneurysm after anastomotic surgery [52].

Several intracranial artery diseases are more prevalent in Asian than non-Asian populations: not only moyamoya disease, but also atheromasic stenosis and intracranial artery dissections. In non-Asians, intracranial dissections account for <10% of all dissections [53], whereas, in Asians, intracranial artery dissections are more common than extracranial artery dissections [15,54]. Genetic variation may partially explain these differences, and RNF213, which is highly prevalent in Asian populations, might be one of the candidate genes to be investigated. In the small cohort described by Kim et al. [33], of the twenty-four patients with intracranial artery dissection, eight (33.3%) had an RNF213 variant. All patients had the same heterozygous p.R4810K (c.14576G>A) variant, with a significantly higher prevalence (*p* = 0.023) in patients with intracranial artery dissection compared to controls. Interestingly, after adjusting for hypertension and RNF213 polymorphism, both hypertension (adjusted odds ratio [OR], 10.185; 95% confidence interval [CI], 1.066 to 97.305; *p* = 0.04) and the presence of the RNF213 variant (adjusted OR, 14.247; 95% CI, 1.563 to 129.841; *p* = 0.018) were independently associated with intracranial artery dissection. In the control group, eight patients with extracranial ICA dissection were included and none had RNF213 variants. Notably, one of the patients with intracranial artery dissection and the RNF213 variant had a family history of moyamoya disease. 

In addition, Kim et al. [36] investigated the relationship between MCA steno-occlusion and RNF213 variants, finding that one of their four patients with intracranial MCA dissection harbored the c.14576G>A variant, but only a minority of the enrolled patients underwent genetic testing: 31 patients, 16/36 (44.4%), in the non-atherosclerosis group and 15/45 (33.3%) in the atherosclerosis group. Thirteen cases were heterozygous for RNF213 variants. RNF213 heterozygotes were more frequent in the non-atherosclerosis than in the atherosclerosis group. It is interesting to note that the diagnostic criteria for moyamoya disease were primarily developed for children and it is not completely known if they might be applied in the same way to adults. In fact, adult-onset moyamoya disease can present with unilateral MCA steno-occlusion [26], progressing over years to bilateral moyamoya disease [55,56]. In addition, a focal MCA stenosis without the sufficient development of moyamoya collaterals at the early stages of adult-onset moyamoya disease has been described [57], and even the initial involvement of the mid-portion of M1 MCA [58]. Shinya et al. [36] reported a case of a patient with an MCA dissection and the RNF213 p.Arg4810Lys, which progressed to involve ICA with a moyamoya disease-like angiogenesis over six years. This case might help to consider some non-atherosclerotic intracranial diseases as a continuum or overlapping entities. The RNF213 p.Arg4810Lys variant could be a common susceptibility factor. It was found in about 80% of moyamoya disease patients in Asia [59], in non-cardioembolic cerebral infarction in the Japanese population [59], in about 2% of the general population in East Asian people as a stroke-related genetic factor [2,4,60,61] and, finally, in several systemic vascular diseases, such as coronary stenosis, pulmonary hypertension and intracranial artery stenosis [11,12,15,61,62,63,64,65].

Tashiro R et al. [34] considered only patients with intracranial VA dissection in comparison with patients with moyamoya disease, using the fragility of the vessels as their mean trait. The authors found the RNF213 c.14576G>A variant in 69.0% (40/58) of the moyamoya disease group, 0% (0/24) of the intracranial VA dissection group and 4.2% (2/48) of the healthy control group. These differences were significant in the adjusted multivariate analysis. In a cohort of patients with intracranial atherosclerosis, Shinya et al. [16] did not find patients with atherosclerotic lesions in the posterior circulation harboring the RNF c.14576G>A variant, suggesting that there is no relationship between this gene and vascular lesions in the posterior circulation.

From an embryological standpoint, there is a theory that views moyamoya disease through the lens of neurocristopathy, suggesting that it primarily affects arteries derived from the neural crest rather than those of mesodermal origin. From this perspective, the RNF213 gene variants are not only observed in moyamoya disease patients but also in those with anterior circulation atherosclerosis rather than posterior circulation [16,33,38]. However, delineating between anterior and posterior circulation can be challenging, as highlighted by Komiyama et al. [66]. Komiyama proposed that moyamoya disease represents a progressive arteriopathy of the primitive ICA, with its pathogenesis being influenced by genetic factors. Notably, he emphasized that the distal cortical branches of the primitive ICA, vertebral artery, basilar artery and external carotid artery remain unaffected, while steno-occlusive changes primarily occur near the bifurcation of the cranial and caudal divisions of the primitive ICA [66]. The existing data regarding the absence of RNF213 variants in posterior circulation atherosclerosis are insufficient to draw conclusive conclusions, especially considering that some arteries in the posterior circulation derive from the neural crest. Komiyama et al. [66] outlined the embryological distribution of neural crest cells, which extends to the territory of the primitive ICA and divides into cranial and caudal divisions at the origin of the posterior communicating artery [67]. The cranial division includes the distal ICA, anterior and middle cerebral arteries and the anterior choroidal artery, while the caudal division comprises the posterior communicating artery, the P1 segment of the posterior cerebral artery, the distal basilar artery and other arteries [68]. During early embryogenesis, significant vascular transformations occur, including the shift of the telencephalic branch of the primitive anterior choroidal artery to the caudal division of the primitive ICA, eventually forming the P2-4 segments of the posterior cerebral artery [67,68,69]. This process results in the dispersion of neural crest cells to various arterial structures, contributing to the formation of the arterial circle of Willis and its branches. The exact embryological border between the primitive internal carotid system and the vertebro-basilar system remains variable. It has been suggested that RNF213, a susceptibility gene for moyamoya disease, may primarily affect derivatives of the primitive ICA among intracranial vessels. However, it is important to note that RNF213 has not been implicated in congenital ICA and MCA anomalies in a Japanese case series. Nevertheless, there have been reports of individuals with RNF213 gene mutations presenting with ruptured MCA peripheral multiple aneurysms associated with twig-like MCA [70].

The hypothesis emerging from these observations is that RNF213 variants may be associated with intracranial artery dissection, potentially increasing the vulnerability of intracranial arteries to dissection. This aligns with the broader role of RNF213 polymorphisms as non-specific markers that increase the vulnerability to intracranial arterial diseases, possibly acting as one of multiple predisposing factors [39]. This hypothesis may partially explain the higher prevalence and risk of intracranial arterial diseases, including intracranial atherosclerosis and artery dissections, in Asian populations [71], where RNF213 variants are more prevalent among East Asians than Caucasians [38]. However, the strength of this association is generally low, and the various limitations outlined in individual studies may affect the final conclusions. One limitation of the general interpretation of the causative role of RNF213 variants in intracranial and systemic arteriopathies lies in the structural properties of this gene. Indeed, RNF213 has a very long open reading frame, encoding a huge protein (length greater than 5000 aminoacyds). Due to these properties, and particularly the length of the gene, rare variants or mutations that affect the protein coding capacity might be expected at a higher rate. This issue should lead to caution in the interpretation of the role of variants of unknown significance.

A final observation might be raised about the association of RNF213 variants with intracranial and systemic vascular disease, including intracranial dissection, moyamoya disease, intracranial aneurisms and visceral stenoses (i.e., renal artery stenosis) and the independent role of arterial hypertension as risk factor for intracranial dissection. This pattern could resemble the known focal form of fibromuscular disease [72], where intracranial involvement apart from aneurysms is rare but has been reported. 

## 5. Conclusions

RNF213 is a gene involved in vascular wall remodeling and documented as a susceptibility marker in several intracranial and systemic vascular diseases, particularly in Asian populations. It might also be involved in intracranial artery dissection and the actual rate ranges from 18.9% to 34.5%, depending on the inclusion or exclusion of VA dissections. All patients had the RNF213 p.Arg4810Lys variant. The small number of patients, the inclusion of only patients of Asian descent and the small but non-negligible coexistence with moyamoya disease familiarity might be limiting factors, requiring further studies to confirm these preliminary findings and the embryological interpretation.

## Figures and Tables

**Table 1:** Data of the included studies on RNF213 testing in intracranial artery dissection.

Reference	Country	Intracranial Artery Dissection Patients (N)	Sex	Age (Years) (Mean ± SD)	Dissected Artery	Diagnostic Technique (N)	Genetic Testing	RNF213 Variants (N/%)	Comments
Kim JS 2018 [33]	Korea	24 cases vs. 24 age and sex matched controls	8 males (33.3%)	41.8 ± 10.2	21 MCA, 2 ICA, 1 PCA	MRA (n = 21), CTA (n = 3), DSA (n = 13), HRMRA (n = 21).	Blood samples for major SNIPs of RNF213 in East Asian patients [2] (p.D4013N, p.P4608S, p.R4810K, p.R4853K, p.D4836N and p.E4950D) amplifying and sequencing three exons (44, 60 and 62)	8 (33.3) in cases vs. 1 (4.2) in controls All had heterozygous p.R4810K (c.14576G>A) variant	11 had ischemic stroke, 7 had TIA, 3 had headache, and 3 pts were asymptomatic
Tashiro R et al., 2019 [34]	Japan	24 patients with intracranial VA dissection, 62 patients with moyamoya disease and 48 healthy controls	NR	NR	Intracranial VA	MRA and BPAS-MRI were used to diagnose intracranial VA dissection [35]	Saliva sample for RNF213 c.14576G>A polymorphism	69.0% (40/58) in the moyamoya disease group, 0% (0/24) in the intracranial VAD group and 4.2% (2/48) in the healthy control group	
Shinya Y 2020 [36]	Japan	1	Woman	63	MCA	MRA DSA	Blood sample	p.Arg4810Lys (rs112735431) variant	Subarachnoid hemorrhage with left MCA dissection resulting in an irregular shape and fusiform dilation of the left M2 MCA wall. Progression to moyamoya disease pattern
Kim YJ 2016 [37]	Korea	4/81 patients had MCA dissection	NR	NR	MCA	MRA HR-MRI [38]	Blood samples for major SNIPs of RNF213 in East Asian patients [2] (p.D4013N, p.P4608S, p.R4810K, p.R4853K, p.D4836N and p.E4950D) amplifying and sequencing three exons (44, 60 and 62)	Only the heterozygote of p.R4810K was more frequent in the non-atherosclerosis group than the atherosclerosis group (62.5% [10/16] versus 26.7% [4/15], respectively; *p* = 0.045).	81 patients < 60 years old with isolated MCA steno-occlusion: 45 (56.6%) in the atherosclerosis and 36 (44.4%) in the nonatherosclerosis Group. 28/36 (77.8%) in non-atherosclerosis group had suspected moyamoya disease

SNIPs: single-nucleotide polymorphisms; MCA: middle cerebral artery; ICA: internal carotid artery; PCA: posterior cerebral artery; SD: standard deviation; TIA: transient ischemic attack; MRA: Magnetic Resonance Angiography; CTA: Computed Tomography Angiography; HRMRA: high-resolution MRA; VA: vertebral artery.

## Data Availability

Not applicable.
